# Cosmic Coincidences: Investigations for Neutron Background Suppression

**DOI:** 10.6028/jres.112.007

**Published:** 2007-04-01

**Authors:** Craig R. Heimbach

**Affiliations:** National Institute of Standards and Technology, Gaithersburg, MD 20899-8461

**Keywords:** background, coincidence, cosmic ray, neutron, muon, special nuclear material

## Abstract

Two experimental investigations were made in order to reduce background counts in neutron detectors. Each investigation relied upon the fact that neutron background is largely due to cosmic ray interactions with the air and ground. The first attempt was to look at neutron arrival times. Neutron events close in time were taken to have been of a common origin due to cosmic rays. The second investigation was similar, but based on coincident neutron/muon events. The investigations showed only a small effect, not practical for the suppression of neutron background.

## 1. Introduction

Neutron background interferes with attempts to measure low neutron levels. The neutron background rate of 0.015 n cm^−2^s^−1^ [1] is sometimes competitive with, and may dominate, the desired neutron signal. This background is typically due to cosmic ray interactions with the atmosphere and ground. This paper reports the results of two attempts to reduce neutron background with coincidence techniques. Both attempts relied upon the original cosmic particle generating a shower of secondary particles. Since each shower has a single triggering event, all the secondary particles must be, to some extent, coincident.

The first background suppression technique involved observing neutron/neutron coincidences. In this case, a single detector can be used to monitor neutrons. If the detection times for two neutrons is within a small time window, the neutrons may be rejected as being due to a single shower. The search was for a time window sufficiently small that few accidental coincidences would occur, but sufficiently large to catch a significant fraction of true coincidences.

The second background technique investigated was to suppress neutron background by detecting coincidences between neutrons and muons. The principle is essentially the same as that for neutron/neutron coincidence, except that a large, efficient muon detector is relatively inexpensive. The attempt was to determine the presence of a cosmic-ray shower with the muon detector, and to reject any neutrons within a given time of the muon. The experiment was flexible enough to detect offset coincidences, where the muon detection might be consistently before or after the neutron event whether because of differences in the particle shower or differences in the electronics.

Even if either of the coincidence techniques worked, further investigations would have to be made into the extent to which the neutron source to be measured might provide coincidences. For example, a spontaneously fissioning nucleus might produce 2 to 3 neutrons in coincidence with gamma rays. Depending on the detector geometry, these might be misinterpreted as cosmic-ray coincidences.

## 2. Neutron/Neutron Coincidences

**Fig. 1:**
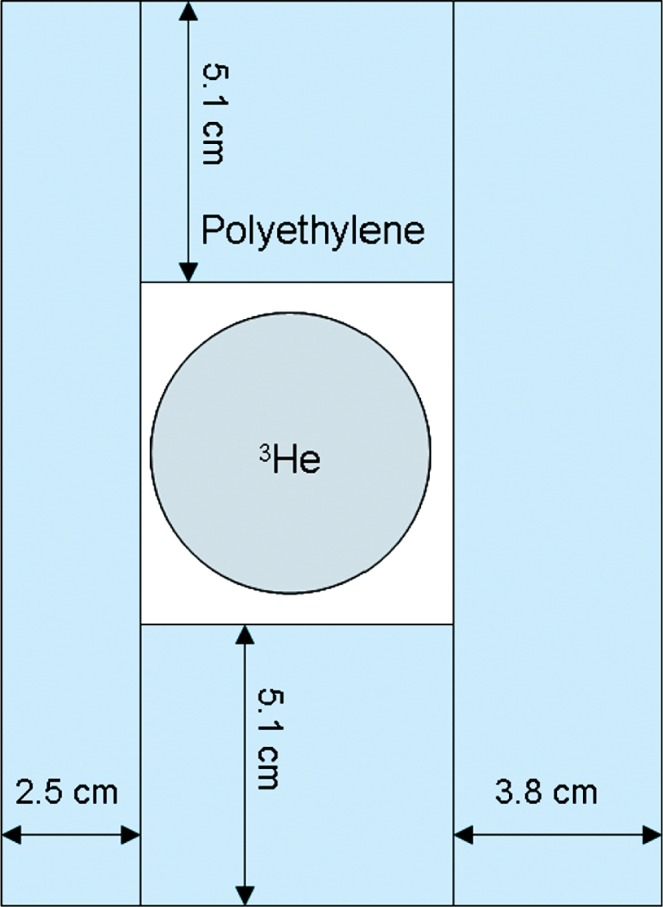
^3^He detector embedded in polyethylene.

**Fig. 2:**
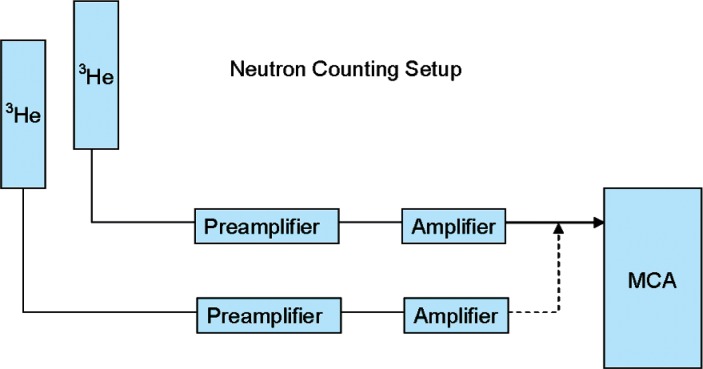
Neutron counting setup. Sometimes only one detector was used.

The detector used for neutron/neutron coincidence measurements was a 178 cm long by 5.1 cm diameter, 304 kPa (3 atmosphere) ^3^He cylinder. The detector was embedded in a polyethylene block 16.5 cm × 12.1 cm × 193 cm with a central square hole to contain the detector. Figure 1 shows a cross section of the geometry. This detector is similar to that used in conventional portal monitors, and was taken from one for this experiment. The detector was operated vertically. Figure 2 shows a block diagram of the data acquisition setup. The background count rate was about 1.3 s^−1^.

^3^He is mainly sensitive to thermal neutrons and relies on the polyethylene to scatter fast neutrons down in energy so that they may be detected. The detector is asymmetric, but remains generally sensitive to neutrons from thermal to about 10 MeV to within about a factor of two. The detector undercounts high-energy neutrons (E_n_ > 10 MeV) because of the thickness of polyethylene. The conclusions derived here may not apply to these high-energy neutrons, but these neutrons are normally of cosmic origin, and may be distinguished from special nuclear material by their energy.

Data were taken with this detector with a multichannel analyzer (MCA) in multiscaling mode. That is, the number of counts from the detector was recorded as a function of time in increments of 5 ms each. Once a set of 8192 time increments was measured, the data were recorded and the data acquisition restarted. This follows the data acquisition model of most neutron monitors. An alternative method of data acquisition, recording the time of each event, was not pursued due to equipment availability. Either method will give similar results.

The 5 ms interval was chosen such that the number of accidental coincidences would be negligible. Also, larger time intervals (10, 15, 20 ms, etc.) could be analyzed from the same data by merging adjacent bins. If a significant number of coincidences were found with the 5 ms time interval, the interval could be reduced in follow-on measurements. If the detector was triggered to reject all counts following within 5 ms of an event, all the 5 ms coincidence events would be rejected at a cost of a 5 ms dead time. At 1.1 s^−1^, this would be about 0.5 % dead time.

**Fig. 3:**
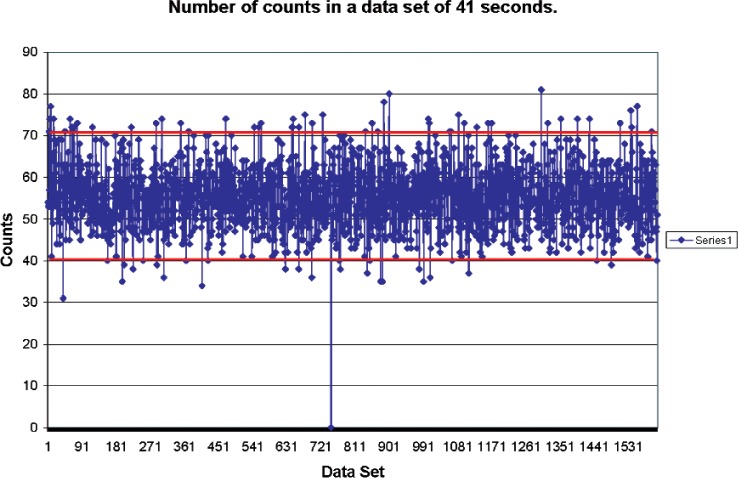
Number of counts taken in 41 seconds in successive counts.

Figure 3 shows a plot of data taken overnight. The data plotted are the sum of the counts from successive multichannel scaling runs, each run consisting of 8192 channels and lasting approximately 41 s. As expected, most of the data are within the two standard deviations plotted with the horizontal lines. One of the channels shows zero counts, however. This is far eyond what might be expected from statistics, and is probably a result of the counting system failing to accumulate counts during that run. Runs with zero counts in a full 8192 bin spectrum were not included in the analysis.

Table 1 breaks this data down into the number of counts per 5 ms bin. The “expected” row is calculated from Poisson statistics using the measured count rate, assuming no correlation among events.

While statistically significant, the number of coincidences is not useful. Even if every excess coincidence could be rejected, the decrease in background count rate would be about 0.2 %. Rejecting all coincidences would reduce the count rate by less than 1 %.

## 3. Alternate Time Scales

For purposes of background rejection, there is no point in detecting coincidences on a finer time scale. Any time increment less than 5 ms would show fewer coincidences than in Table 1, even though the coincidences would be more likely to be real.

The same data were analyzed in time increments of 10, 20, 50, and 100 ms. In each case there was a measurable, but small, coincidence effect. Table 2 shows the same data as in Table 1 rebinned into 100 ms intervals by merging successive bins. As with the shorter time scale, there is a statistically significant time correlation among neutron events, but not high enough to reject a useful number of background neutrons.

More efficient and larger detectors might be expected to have a higher coincidence rate. For example, if all events produced multiple neutrons randomly over an area A and the detector had an effective sensitive area S, then the detector would measure S/A of the events. For a given measured event, there would be a probability of S/A that a true additional neutron (not accidental coincidence) would be detected. For the 5 ms intervals in Table 1, there were 146 excess coincidences in 88608 bins with counts, for a S/A of 0.0016. For 100 ms intervals, S/A is 0.0020. A 50 % background rejection rate would require an S/A of 0.5, or approximately 300 times the detection sensitivity as measured. This represents an optimistic limit since it ignores neutrons arriving as true singles.

Table 3 shows results for two ^3^He detectors, each identical to the detector used for the data in Tables 1 and 2. The detectors were separated by about 5 meters. For these results, both detectors were connected to the same multichannel scaling counter, so the two detectors were acting as a single large detector. The S/A value for this system was 0.0017, only marginally higher than the 0.0016 from the single-detector data. This indicates that a high fraction of neutrons reach the ground as singles.

## 4. Neutron/Muon Coincidences

Even if the neutrons arrive as singles, the possibility that they might arrive in coincidence with other types of particles was explored. In particular, the cosmic-ray-induced muons are much easier to detect than neutrons. The possibility that neutron/muon coincidence detection could help reject background neutrons was explored.

**Fig. 4:**
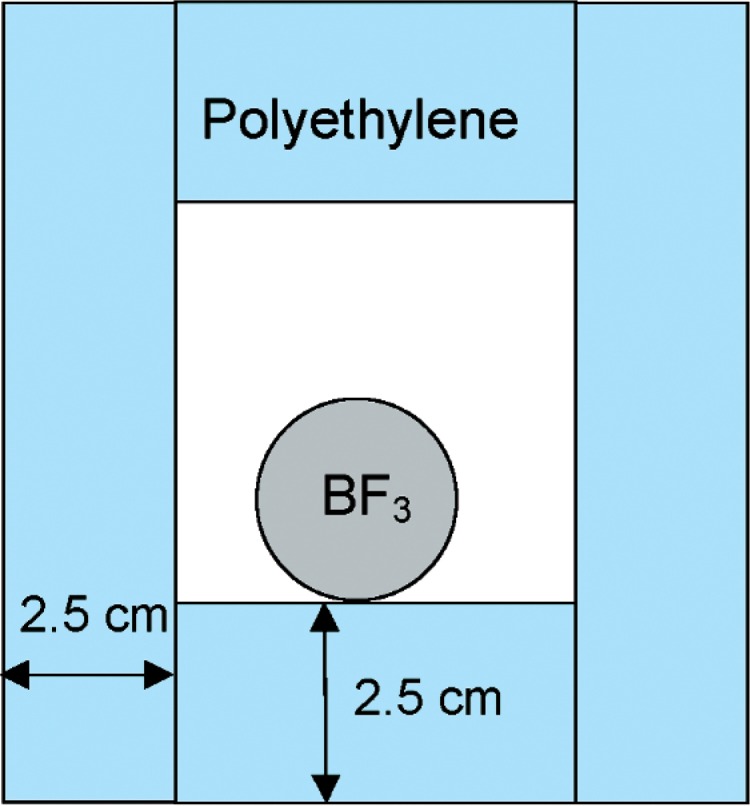
BF_3_ detector embedded in polyethylene.

A separate detector system was developed to measure neutrons in coincidences with muons. The neutron detector used in the first part of the experiment was not available, so a similar, but smaller, boron trifluoride (BF_3_) system was developed. This used a 2.5 cm diameter by 26.7 cm BF_3_ tube. The length of the tube was surrounded by 2.5 cm of polyethylene along its length, with a 5.1 cm square inner void for the detector. Figure 4 shows a cross section of the detector/polyethylene system.

**Fig. 5:**
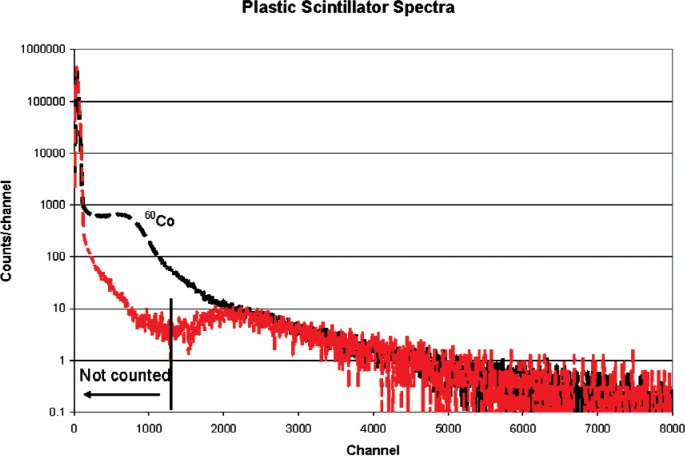
Background (muon only) and ^60^Co spectra.

The muon detector was a plastic scintillator, Bicron type BCF-412, 1 m square and 2 cm thick. The thickness was chosen to give muons a higher pulse height than background gamma rays. Figure 5 shows a scintillator background spectrum and a spectrum measured with a ^60^C source near the scintillator. For coincidence measurements, only pulses higher than the midpoint of the valley (to the right of the vertical line in Fig. 5) were counted. These correspond to about 1.7 MeV in terms of gamma-ray energy.

**Fig. 6:**
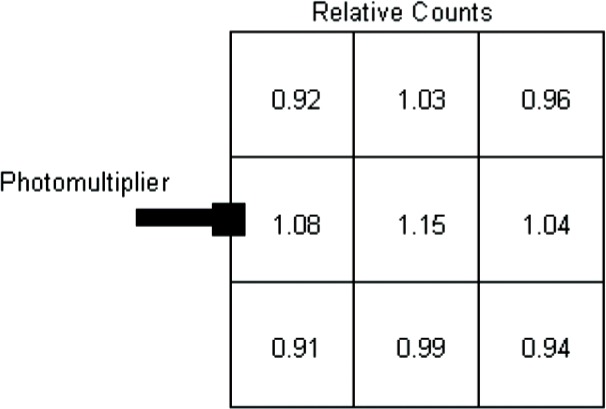
Relative ^60^Co sensitivity as a function of position.

The muon paddle had a single photomultiplier mounted in the center of one edge. Figure 6 shows the measured relative sensitivity of each section. The sensitivity was measured by placing a ^60^C source in the center of each of the nine sections.

Both the neutron detector and the muon detector were horizontal for the measurements.

**Fig. 7:**
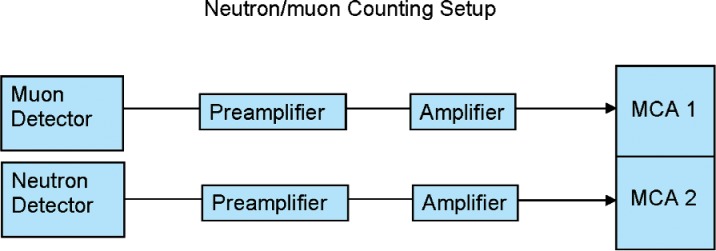
Neutron/muon block diagram.

The coincidence system was similar to that in the first part of the experiment, except that two synchronized multichannel scalers were used. The pulses from each detector were stored in a separate multichannel analyzer in multichannel scaling mode. The analyzers were synchronized so that a pulse in each analyzer at the same time appeared in the corresponding channel of each analyzer. See Fig. 7.

In the analysis, adjacent channels were also scanned to see whether delayed coincidences occurred. For example, if the neutron parts of the shower consistently occurred after the muon portions, there could be coincidences with a time offset to allow for the delay between the neutron and muon portion of the shower. The data acquisition time increment was shortened from 5 ms to 1 ms because of the high muon count rate. 1 ms reduced accidental coincidences. At a muon count rate of about 150 s^−1^ above the threshold, there was about a 15 % chance of a muon count in any time bin.

**Fig. 8:**
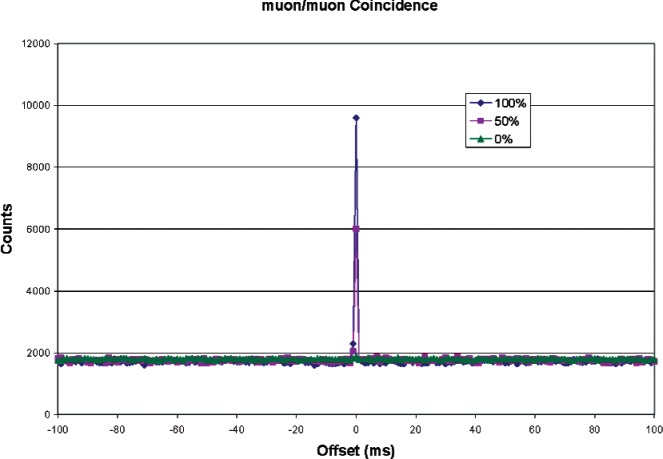
Muon/muon coincidence rate as a function of paddle overlap.

**Fig. 9:**
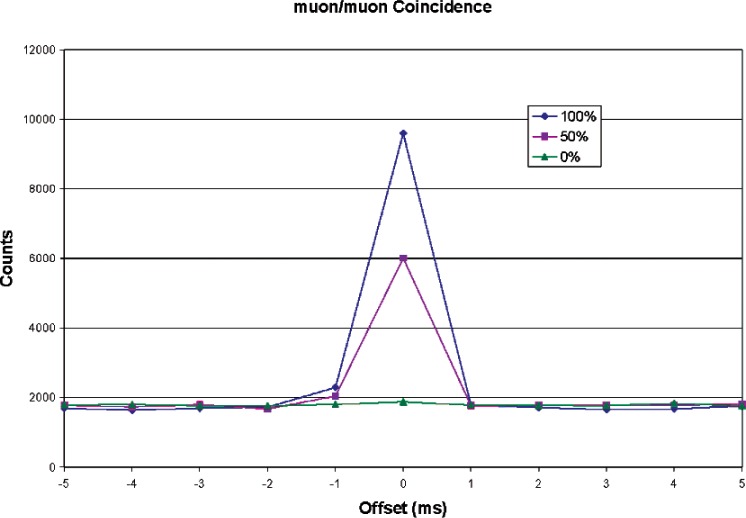
Muon coincidence rate, expanded view.

As a test of the system, identical muon paddles were used instead of muon and neutron detectors. Figure 8 shows coincidence results with one paddle directly above the other (100 % overlap), one paddle above the other but offset by 50 cm (50 % overlap), and the two paddles side-by-side (0 % overlap). Data increments were 1 ms. Figure 9 shows the same data, but expanded around time zero (true coincidence). The figures show a high coincidence rate with 100 % paddle overlap, and decreasing as the overlap went to zero. With one paddle over the other, approximately 85 % of the pulses in either channel were in coincidence with a pulse from the other. For 50 % overlap, the coincidence rate was 49 %. With no overlap, the coincidence rate was still about 1 % above background.

**Fig. 10:**
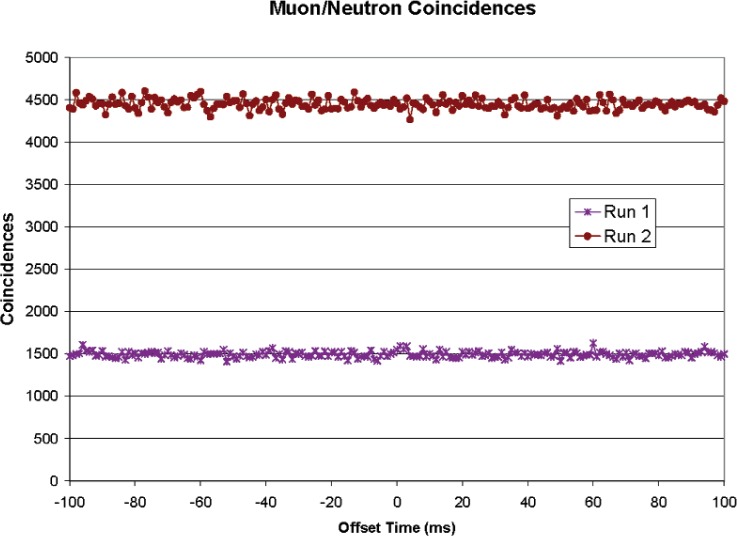
Muon/neutron coincidence data.

The low level of muon/muon coincidence did not promise much for muon/neutron coincidences, but the data were taken. Figure 10 shows the results. The possibility of delayed coincidences was checked out to 100 ms. Any true coincidences would show as a peak, as in Fig. 8, among a background of accidental coincidences. Run 1 gave a hint of a peak at about 2 ms, but this was not confirmed in a second run. The reason for the different average number of coincidences between the two runs is that the second run was about three times as long as the first.

For the first set of data in Fig. 10 (Run 1), there are 1493 counts in each channel, with a statistical fluctuation of about ± 37 (1σ) counts each. Since there are 201 channels, one would expect a few 2σ deviations by chance. The largest deviation is 3.7σ, in channel 63. This is consistent with no correlations. A true correlation rate of 0.015 (1.5 % of neutrons having a coincident muon) would give a peak of 4σ on the average.) A true coincidence rate of 0.025 would produce a 4σ peak 95 % of the time and will be used as the upper limit of coincidence consistent with the first run.

The second run, three times as long, also shows no peak and limits the true coincidence rate to 0.015.

## 5. Calculational Analysis

A set of Monte Carlo calculations was performed in order to put some of the above results into context. MCNPX [2] was used to perform the calculations.

The model was of a flat earth with a point proton source, directed straight down, directly above the detector which was at ground zero. The source was a high energy proton spectrum taken from Greider [3]. The atmosphere was of variable density out to 50 km. The tallies were F4 (fluence) tallies. These calculations did not model reality, especially in the angular distribution of incident particles, but were selected to give insight into the measurements.

Unfortunately, MCNPX does not have the capability of computing coincidence reactions for many of the particles, so the results of the experiment could not be calculated directly.

**Fig. 11:**
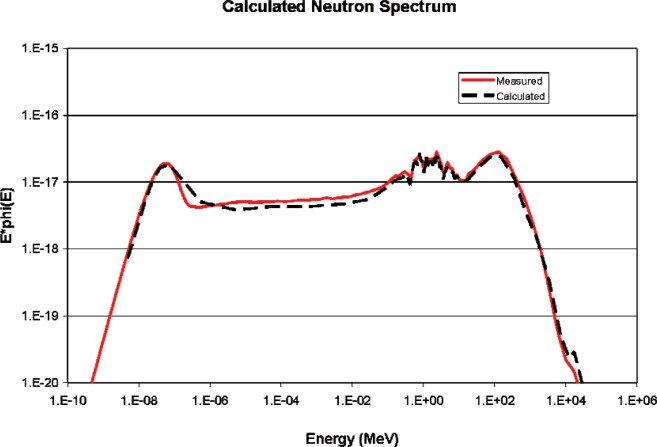
Measured [1] and calculated neutron spectra.

**Fig. 12:**
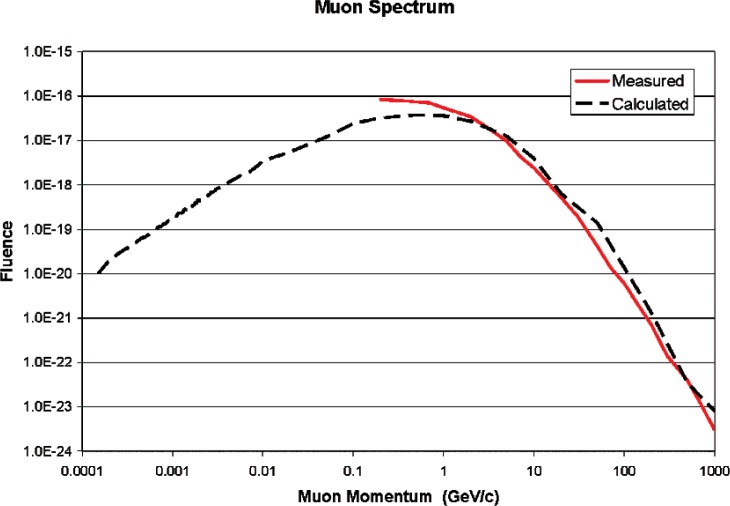
Measured [4] and calculated muon spectra.

Figures 11 and 12 show the calculated neutron and muon spectra compared against the referenced measurements [1,4]. Agreement between measurement and calculation tends to confirm the utility of the model.

**Fig. 13:**
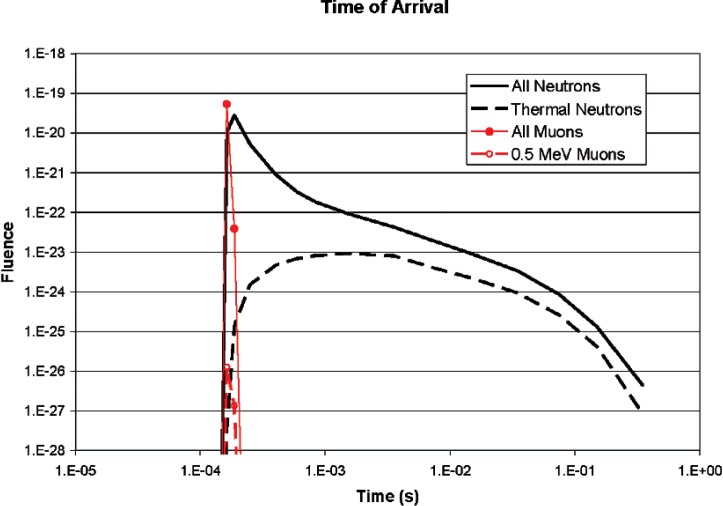
Times of arrival of neutrons and muons.

**Fig. 14:**
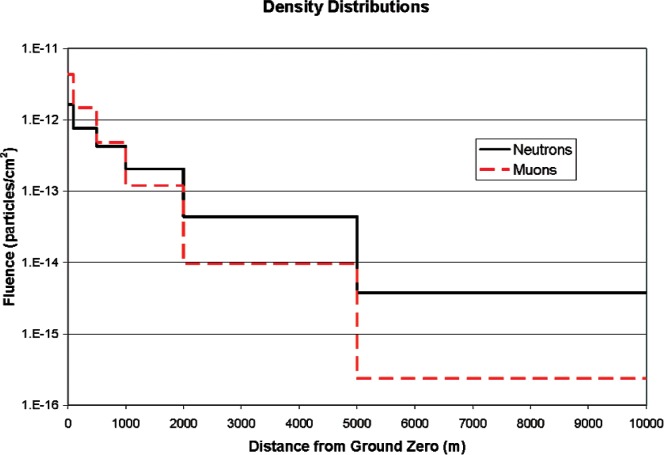
Neutron and muon fluence density as a function of distance from ground zero.

Figure 13 shows the times of arrival of the neutron and muon showers. The neutrons arrive after the muons, but the bulk of them arrive within the 100 ms time window used for the measurements in this experiment. The muons arrive in a bunch, traveling essentially at the speed of light. The neutrons are more spread out in time, making either neutron/neutron or neutron/muon coincidence peaks much wider than if they all arrived simultaneously. In the neutron/neutron data discussed above, the 100 ms reanalysis of the data would have shown any significant correlation, if present. In the muon/neutron data, this would result in a wide peak, offset to the left, in Fig. 10. The ratio of the number of events in which a muon count occurred within 100 ms before the neutron by the number of events in which a count occurred within 100 ms after the neutron is 0.997 for run 1 and 1.004 for run 2. Figure 14 shows the muon and neutron fluence density as a function of distance from the point on the ground directly below the source (ground zero). Both types of particles are bunched together near ground zero. This would tend to increase coincidences measured at the same location. Half the neutron fluence is within 2 km of ground zero. The muons are more tightly bunched, with half within 900 meters of ground zero. In either case, the spot size is large compared to the detector size.

## 6. Conclusions

Measurements were made of neutron/neutron and of neutron/muon coincidences in an attempt to identify neutrons of cosmic origin. These neutrons dominate the neutron background. Neutron/neutron coincidences were found, but not in sufficient quantities to be useful for background suppression. Neutron/muon coincidences were not found at the 1.5 % level. Contributing factors to the lack of coincidences measurements were the arrival of uncorrelated neutrons, their spread in arrival time, and the large area in which they are distributed. Even muon/muon events, which are more likely to be found in coincidence, showed only a small correlation, even with a larger and more efficient detector than normally available for neutrons.

## Figures and Tables

**Table 1:** ^3^He counts binned into 5 ms intervals

Measured/Expected	Bins with 0 counts	Bins with 1 counts	Bins with 2 counts	Bins with 3 counts	Bins with 4 counts	Bins with 5 counts
Measured	13 084 128	88 162	437	9	0	0
Expected	13 083 973	88 463	299	0.6740	0.0011	1.54E −06
Measured/Expected	1.000	0.997	1.461	13.353	0.000	0.000
Measured Excess	154.6	− 300.9	137.9	8.3	0.0	0.0

**Table 2:** ^3^He counts binned in 100 ms intervals

Measured/Expected	Bins with 0 counts	Bins with 1 counts	Bins with 2 counts	Bins with 3 counts	Bins with 4 counts	Bins with 5 counts
Measured	574 696	77 311	5375	277	12	1
Expected	574 479	77 694	5254	236.842	8.008	0.217
Measured/Expected	1.000	0.995	1.023	1.170	1.499	4.617
Measured Excess	216.7	−382.8	121.3	40.2	4.0	

**Table 3:** ^3^He counts from two detectors, 5 ms intervals

Measured/Expected	Bins with 0 counts	Bins with 1 counts	Bins with 2 counts	Bins with 3 counts	Bins with 4 counts	Bins with 5 counts
Measured	44 204 086	287 972	1428	26	0	0
Expected	44 208 987	289 010	945	2	0	0
Measured/Expected	1.000	0.996	1.512	12.630	0.000	
Measured Excess	− 4900.8	− 1038.4	483.3	23.9	0.0	0.0
